# Evaluating macroscopic sex estimation methods using genetically sexed archaeological material: The medieval skeletal collection from St John's Divinity School, Cambridge

**DOI:** 10.1002/ajpa.23753

**Published:** 2018-12-21

**Authors:** Sarah Inskip, Christiana L. Scheib, Anthony Wilder Wohns, Xiangyu Ge, Toomas Kivisild, John Robb

**Affiliations:** ^1^ McDonald Institute for Archaeological Research, University of Cambridge Cambridge United Kingdom; ^2^ Institute of Genomics, University of Tartu Tartu Estonia; ^3^ Big Data Institute, University of Oxford Oxford United Kingdom; ^4^ Faculty of Biology, Medicine and Health, School of Biological Sciences, Division of Musculoskeletal and Dermatological Sciences University of Manchester Manchester United Kingdom; ^5^ Department of Archaeology University of Cambridge Cambridge United Kingdom

**Keywords:** genetic sex, medieval, preauricular sulcus, sex estimate accuracy

## Abstract

**Objectives:**

In tests on known individuals macroscopic sex estimation has between 70% and 98% accuracy. However, materials used to create and test these methods are overwhelming modern. As sexual dimorphism is dependent on multiple factors, it is unclear whether macroscopic methods have similar success on earlier materials, which differ in lifestyle and nutrition. This research aims to assess the accuracy of commonly used traits by comparing macroscopic sex estimates to genetic sex in medieval English material.

**Materials and Methods:**

Sixty‐six individuals from the 13th to 16th century Hospital of St John the Evangelist, Cambridge, were assessed. Genetic sex was determined using a shotgun approach. Eighteen skeletal traits were examined, and macroscopic sex estimates were derived from the os coxae, skull, and os coxae and skull combined. Each trait was tested for accuracy to explore sex estimates errors.

**Results:**

The combined estimate (97.7%) outperformed the os coxae only estimate (95.7%), which outperformed the skull only estimate (90.4%). Accuracy rates for individual traits varied: Phenice traits were most accurate, whereas supraorbital margins, frontal bossing, and gonial flaring were least accurate. The preauricular sulcus and arc compose showed a bias in accuracy between sexes.

**Discussion:**

Macroscopic sex estimates are accurate when applied to medieval material from Cambridge. However, low trait accuracy rates may relate to differences in dimorphism between the method derivative sample and the St John's collection. Given the sex bias, the preauricular sulcus, frontal bossing, and arc compose should be reconsidered as appropriate traits for sex estimation for this group.

## 1 INTRODUCTION

Estimating biological sex from human skeletal remains is fundamental to most bioarchaeological research (Brickley & Buckberry, 2017). As there are differences in the growth, development, form, and senescence between the sexes, knowledge of an individual's sex is usually required prior to analysis of other biological features, including age, stature, and disease presence. Furthermore, in many societies biological sex is important in gender construction which is often a key aspect of social organization (Sofaer, 2005). While macroscopic methods currently used to estimate sex in archaeological remains are accurate when tested on postmedieval (1485–1800) and modern (1800 onwards) known‐sex individuals (e.g., Đuric, Rakočević, & Đonic, 2005; Lewis, Heather, & Gavin, 2016; Listi & Bassett, 2006; Mays & Cox, 2000; Meindl, Lovejoy, Mensforth, & Don Carlos, 1985; Thomas, Parks, & Richard, 2016; Ubelaker & Volk, 2002; Williams & Rogers, 2006), it could be argued that this high level of accuracy may reflect the fact that the populations tested are temporarily similar, or even the same collections used to create the methods.

Concerns over the relevance of trends drawn from post‐/industrial samples to material from other periods and locations has been raised (Ubelaker, 2008; Walker, 2008), because of the fact that sexual dimorphism varies between groups due to differences in growth and development, disease (Ubelaker & DeGaglia, 2017), activity patterns (Krishan et al., 2016), general secular trends (Godde, 2015), and genetic admixture. This issue is highlighted in research where some methods or sexually dimorphic features appear accurate in one population, but not for others (e.g., Maat, Mastwijk, & Van der Velde, 1997; MacLaughlin & Bruce, 1990; Spradley & Jantz, 2001; Walker, 2005). Specifically, in the development of sex estimation standards 20th century individuals have been heavily utilized (e.g., the Herman‐Todd, William Bass, and the Terry collections), which also includes war‐dead samples (e.g., Korean War and Balkan conflict) that are biased toward a selected groups of individuals either by sex, or fitness to serve in the military. Both differ significantly in lifestyle and nutrition to preindustrial communities. Even for modern and postmedieval samples, there are discrepancies in accuracy rates (see Table 1). As such, it is not entirely clear how accurate commonly used methods/traits as assessed on recent material are for estimating sex for skeletal material predating the industrial period.

**Table 1:** Examples of published accuracy rates on known‐sex and age skeletal collections

Source	Skull	Os coxae	Skull and os coxae
Meindl et al. (1985)	92.0%	96.0%	97.0%
Đuric et al. (2005)	70.6%^a^	93.5%	100.0%
Molleson and Cox (1993)	–	–	98.0%
Williams and Rogers (2006)	92.0%	–	–
Lewis et al. (2016)	96.9%	–	–
Lovell (1989) (Phenice traits)	–	83.0%	–
Thomas et al. (2016)	92.0%	96.0%	97.4%
Listi and Bassett (2006)	–	95.0% ^a^	–
Ubelaker & Volk (2002)	–	96.5%	–

a Accuracy rate when ambiguous scored as incorrect.

One of the reasons that accuracy testing has not taken place is the lack of large skeletal collections of known individuals dating prior to the 18th century. However, in recent years ancient DNA (aDNA) testing has revolutionized bioarchaeological research, and has become widely used for sex estimation in archaeological material (Faerman et al., 1995; Stone, Milner, Pääbo, & Stoneking 1996; and more recently Álvarez‐Sandoval, Manzanilla, & Montiel, 2014; Inskip et al., 2015). If aDNA is sufficiently preserved and amplified genetic approaches, when used individually or in combination, have demonstrated 100% accuracy in tests on known‐sex individuals (Daskalaki, Anderung, Humphrey, & Götherström, 2011; Skoglund, Storå, Götherström, & Jakobsson, 2013). Earlier methods include PCR approaches that target a single locus with X‐Y homology, such as the amelogenin gene (Daskalaki et al., 2011), or a set of YSTR loci (Tierney & Bird, 2014). More recent approaches are based on the assessment of shotgun sequence reads mapping to Y versus X chromosome (Skoglund et al., 2013). The success of this method is based on the fact that the vast majority of individuals will have either two X chromosomes (female) or one X and one Y (male), and using a shotgun sequencing approach it is possible to calculate the ratio of reads mapping to the X and Y chromosomes to estimate sex. However, it is important to point out that while the accuracy rates are high, genetic sex is not binary, and it is possible for an individual to be chimeric (have two distinct genomes, which can be of different sex, as a result of the aggregation of two fertilized eggs in utero). These individuals would have an Y/X ratio intermediate to male and female. Individuals with chromosomal copy disorders such as Turner Syndrome (X0), Trisomy X (XXX), Klinefelter Syndrome (XXY), or Jacob's Syndrome (XYY) may also have intermediate Y/X ratios. However, such cases are rare and present in only 1 in 4,500 individuals today (Hughes, Houk, Ahmed, Lee, & LWPES1/ESPE2 Consensus Group, 2006). Although not impossible, this situation is unlikely in random archaeological sample. Importantly, if such a syndrome is present, it has been shown that it is possible to detect all of these abnormalities using a shotgun approach even in the case of very low target DNA against a high contaminating background (Mazloom et al., 2013). Other limitations include structural variation, such as deletions, which can lead to false negative identifications of male sex in methods based on single or small number of loci; it is also difficult to control for contamination in case of PCR based methods; and all genetic methods of sex estimation are limited by the preservation of ancient DNA. Overall, this does mean that aDNA methods still produce an estimate of sex (see Ainsworth, 2015 for summary on genetic sex and its nonbinary nature). Nevertheless, as aDNA accuracy is still higher than for macroscopic methods, it provides a valuable base‐line method for which to assess macroscopic methods.

As a result of improvements in aDNA methodology, computing power and declining costs, many large scale projects have produced genetic sex estimates (e.g., Altena, Smeding, & Knijff, 2013). This permits an assessment of the accuracy of macroscopic sex estimation methods for preindustrial populations, as has been done for forensic cases (Thomas et al., 2016). This will provide valuable information on the levels of sexual dimorphism for specific traits, which would be significant for improving macroscopic sex estimation methods. Such research is essential as although the cost of aDNA analysis is declining, it remains expensive enough to preclude routine analysis by archaeologists, especially in areas where funding for skeletal research is limited, or where aDNA preservation is poor.

This article aims to explore the accuracy of macroscopic traits and combinations of traits used for sex estimation when applied to a skeletal assemblage predating the postmedieval period. This will be achieved by independently estimating sex using macroscopic traits and genetic analysis on the skeletal material from the Hospital of St John the Evangelist, Cambridge (13th–16th century) (here forth known as St John's).^1^ By comparing sex estimations obtained from macroscopic individual traits and combinations of traits with the genetic estimates obtained from shotgun sequencing, accuracy rates for the former will be calculated. In addition, assessing males and females separately will highlight sex biases in accuracy which may result from variation in sexual dimorphism between the St John's collection and the reference populations from which the methods originated. For example, if there is higher population robusticity generally, all males would be sexed correctly by default, but females would be misclassified. Overall, this work, which will be the first large scale analysis of the accuracy of sex estimation traits in medieval populations, will test whether current macroscopic sex estimation methods are successful on a preindustrial population from northwestern Europe. By scrutinising the accuracy of current approaches/traits this research contributes significant knowledge that can improve sex estimation through highlighting which traits should be included or excluded in analyses, and where the cut off values for male and female should be for our population.

## 2 MATERIALS

From 2005 to 2012 excavations at St John's College, Cambridge, revealed a large medieval cemetery belonging to the Hospital of St John the Evangelist. Over 400 well‐preserved articulated skeletons were excavated (Cessford, 2015). As part of the “After the Plague project,” which aims to explore the long term biological impact of the Black Death epidemic of 1348‐50 in Cambridge, 66 adult individuals were selected for aDNA analysis based on the presence of analyzable teeth. This included males and females of all ages (see Table 2), and five unknown age adults. Skeletal completeness varied from 25% to 100%. As there are more males buried at St John's Hospital cemetery, probably because of the fact that the hospital received “poor scholars” and these could only be men, more males were available for genetic testing.

**Table 2:** Age and genetic sex of the adult individuals included in this study

Age range in years	Female	Male	Sex not assigned	Total
Unknown adult	2	3	0	5
15–18	0	1	0	1
18–25	5	7	1	13
25–35	4	3	0	7
35–45	6	5	0	11
45–60	5	13	0	18
60+	3	8	0	11
Total	25	40	1	66

*Note*. Young = 15–25 years, middle = 25–45 years, mature = 45–65 years, old = 60 years+.

## 3 METHODS

### 3.1 Ancient DNA sampling, extraction, and pre‐PCR processing

Samples for aDNA were taken from tooth roots. While wearing gloves to prevent contamination, one tooth from each individual was extracted and placed in a sterile bag. Whole samples from tooth roots were taken with a new disposable circular Dremel wheel attachment (409) in a class II hood at the ancient DNA laboratory at the Department of Archaeology at Cambridge University. Compact root portions were soaked in 6% w/v bleach for 10 min, then rinsed twice with ddH_2_O, soaked in 75% ethanol for 2 min, transferred to a clean paper towel on a rack inside the cleaned drill hood, UV irradiated for 50 min on each side, and then allowed to dry before being weighed and transferred to PCR clean 5 or 15 ml conical tubes for chemical extraction. To extract aDNA, per 100 mg of each sample, 2 ml of 0.5M EDTA Buffer pH8.0 (Fluka) and 50 μl of Proteinase K 10 mg/ml (Sigma Aldrich) were added modified from Dabney et al. (2013). Tubes were rocked in an incubator for 72 hr at room temperature. Extracts were concentrated to 250 μl using Amplicon Ultra‐15 concentrators with a 30 kDa filter (Millipore). Samples were purified according to manufacturer's instructions using the Minelute™ PCR Purification Kit (Qiagen) with High‐Volume spin columns (Roche); samples were incubated with EB at 37 °C for 10 min prior to elution in 50 μl.

### 3.2 Library amplification

Library preparation was conducted using a protocol modified from the manufacturer's instructions included in the NEBNext® Library Preparation Kit for 454 (E6070S, New England Biolabs, Ipswich, MA) as detailed in Sánchez‐Quinto et al. (2012). DNA fragmentation step of the commercial library preparation protocol was omitted and reactions were scaled to half volume; adaptors were made as described in Meyer and Kircher (2010) and used in a final concentration of 2.5 μM each. DNA was purified on MinElute columns with PB buffer (Qiagen, Germany).

Libraries were amplified using the following PCR set up: 50 μl DNA library, 1X PCR buffer, 2.5 mM MgCl_2_, 1 mg/ml BSA, 0.2 μM inPE1.0, 0.2 mM dNTP each, 0.1 U/μl HGS Taq Diamond, and 0.2 μM indexing primer. Cycling conditions were: 5 min at 94 °C, followed by 18 cycles of 30 s each at 94 °C, 60 °C, and 68 °C, with a final extension of 7 min at 72 °C. Amplified products were purified using MinElute columns and eluted in 35 μl EB (Qiagen, Germany). Samples were quantified using Quant‐iT™ PicoGreen® dsDNA kit (P7589, Invitrogen™ Life Technologies) on the Synergy™ HT Multi‐Mode Microplate Reader with Gen5™ software.

Samples were pooled in equimolar amounts and sequenced on the Illumina NextSeq500 platform, with a 75‐cycle single‐end run setting at the University of Cambridge Biochemistry DNA Sequencing Facility. Sequences were returned in the form of four compressed FASTA.GZ files per sample, which were downloaded from Illumina BaseSpace and analyzed on the Estonian Biocentre's server.

### 3.3 Mapping and genotyping

Adapters were removed using CutAdapt (Martin, 2011). Trimmed reads were mapped to hg19 build 37.1 using bwa v0.6.1 (Li & Durbin, 2009). Files were converted to the BAM format for use with SAMTools v1.19 (Li et al., 2009). Duplicate reads were removed using Picard Tools MarkDuplicates (http://broadinstitute.github.io/picard).

### 3.4 Authenticity of results, contamination estimates, and error rates

Damage patterns of bam files were analyzed by MapDamage2.0 (Jónsson, Ginolhac, Schubert, Johnson, & Orlando, 2013 *––* https://ginolhac.github.io/mapDamage). The rate of over>10% C–T transitions in the ends of 5′–3′ reads and G–A in the 3′–5′ reads and the distribution of fragment lengths less than 200 bp is consistent with degradation found in ancient DNA (Briggs et al., 2007; Pääbo, 1989).

Contamination rates were estimated using the principle that known polymorphic sites on haploid genomes and their adjacent sites should have the same error rate unless some modern human contamination is present. Thus, in its simplest form, the mismatch rate in adjacent sites can be subtracted from known polymorphic sites to estimate the contamination rate. Rates of contamination were estimated on mitochondrial DNA by calculating the percentage of nonconsensus bases at haplogroup‐defining positions as utilized in Scheib et al. (2018). Each sample was mapped against the RSRS downloaded from phylotree.org and checked against haplogroup‐defining sites for the sample‐specific haplogroup.

Error rates were estimated using the second available ANGSD method, which uses an outgroup (Chimp) and an “error free” individual, in this case a high coverage, high quality CEU individual downloaded from the ANGSD github repository. The method is covered in detail in the publication of Rasmussen et al. (2011). We followed default parameters listed at http://www.popgen.dk/angsd/index.php/Error_estimation.

### 3.5 Molecular sex estimation

The sex of individuals was estimated using a script by Skoglund et al. (2013) available by download online (https://github.com/pontussk/ry_compute). This script makes use of the ratio of reads mapping to the Y chromosomes over the number of total reads mapping to X and Y (*R*_y_). Ratios of 0.075 or higher indicate males. It was run with default settings as suggested by Skoglund's documentation. Results are either returned as XX or XY, (“XX” when *R*_y_ + CI [=1.96*SE] < 0.016 and “XY” when *R*_y_ − CI >0.075), “consistent with XX but not XY” (*R*_y_ − CI < 0.016 and *R*_y_ + CI < 0.075), “consistent with XY but not XX” (*R*_y_ − CI > 0.016 and *R*_y_ + CI > 0.075), else “Not Assigned.”

### 3.6 Macroscopic sex estimation methodology

Results of aDNA testing were not known until after macroscopic sex estimates were complete. Eighteen skeletal traits were selected for sex estimation based on their presence in widely used standards applied to remains from the British Isles (see Table 3 for a list of traits used and their published sources). Phenice traits, forehead inclination, frontal bossing, and overall mandible shape were scored on a scale of 1–3 (female, unknown, and male). Traits outlined in Buikstra and Ubelaker's (1994) standards and Brickley (2004) were scored on a scale of 1–5 following the standards (female, probable female, unknown, probable male, and male). Four Workshop of European Anthropologists (Ferembach et al., 1980) traits were also scored on a five‐point scale but from −2 (hyperfeminine) to 0 (neutral) to +2 (hypermasculine). These scores were translated to 1 (hyperfeminine) to 5 (hypermasculine), respectively to match the Buikstra and Ubelaker (1994) scale. The only exception was the preauricular sulcus which is scored as present or absent, in which scores 1–4 are a positive confirmation of the trait, with only score 5 (absent) being indicative of “maleness”. In total, 63 individuals could be assessed for macroscopic sex estimation. Intraobserver tests were not carried out as previous intraobserver tests have shown that scoring of traits is reproducible (e.g., Novak, Schultz, & McIntyre, 2012; Walker, 2005, 2008; Williams & Rogers, 2006).

**Table 3:** Os coxae and skull traits tested for accuracy in comparison to aDNA estimations

Os coxae traits	Source
Ventral arc	Phenice (1969)
Ischiopubic ramus ridge	Phenice (1969)
Subpubic concavity	Phenice (1969)
Preauricular sulcus	Buikstra and Ubelaker (1994)
Sciatic notch	Buikstra and Ubelaker (1994)
Subpubic angle	Brickley (2004)
Arch compose	Ferembach, Schwidetzky, and Stloukal (1980)
Iliac shape	Ferembach et al. (1980)
Skull traits	
Supraorbital margin	Buikstra and Ubelaker (1994)
Mastoids	Buikstra and Ubelaker (1994)
Glabella	Buikstra and Ubelaker (1994)
Mental eminence	Buikstra and Ubelaker (1994)
Nuchal crest	Buikstra and Ubelaker (1994)
Frontal bossing (eminences)	Schwartz (2007)
Overall mandible shape	Brickley (2004)
Gonial flaring	Ferembach et al. (1980)
Forehead inclination	Ferembach et al. (1980)
Zygomatic arch extension	Keen (1950)

After individual traits were scored, an estimate was given to the os coxae and skull. This was done based on an average of scores obtained. However, for the os coxae, if the sex estimates returned ambiguous but Phenice traits had given male or female scores, the estimate was revised to take into account the proven higher accuracy of the Phenice traits. An overall sex estimate was given to each skeleton based on all of the traits assessed. Where the general skull and os coxae estimates provided conflicting results, more weight was placed on pelvic traits, as these have been shown to be more reliable (see Table 1). As the material is archaeological in nature, not all traits were observable for all individuals. Although this is an unavoidable factor when dealing with such collections, the results will provide an opportunity to explore the impact of incompleteness on sex estimation, something that is important given the known impact that incomplete suites of traits can have on final results (see Kjellstrom, 2004).

### 3.7 Methodology for comparing aDNA and macroscopic sex estimation

Results from macroscopic and genetic analysis were compared. First we present accuracies of the combined estimate, then the separate os coxae and skull estimates, and finally individual traits. To assess the significance of results two tests were carried out. McNemar tests assessed for systematic differences between the results of the aDNA and macroscopic approaches, whereas Cohen's Kappa tests explored the agreement between the expected (aDNA) and observed (macroscopic) values. This latter is important as it takes into consideration the binary nature of the data and the fact that chance agreement alone could be 50%. Levels of agreement followed those outlined in Watson & Petrie (2010, p. 1170): Poor if *k* < 0.00, Slight if 0.00 ≤ *k* ≤ 0.20, Fair if 0.21 ≤ *k* ≤ 0.40, Moderate if 0.41 ≤ *k* ≤ 0.60, Substantial if 0.61 ≤ *k* ≤ 0.80, and Almost perfect if *k* > 0.80.

In order to improve the accuracy of future sex estimation in our population it was necessary to explore where misclassification occurred. To do this we investigated male and female accuracy rates separately, and assessed the distribution of male and female scores in relation to the standards (where 1 and 2 represent female, 3 ambiguous, 4 and 5 male). We calculated the probability that an individual assigned specific score was either male or female for each trait following Walker (2008, p. 42). This highlights where cut‐off values for male and female should be. In addition, although our sample is not large enough to be broken down into age groups for statistical analysis, attention was paid to the ages of individuals with incorrect sex estimates for overall estimates and traits.

It has been noted that some accuracy rates from sex estimation tests are misleading. McFadden and Oxenham (2015) demonstrated how accuracy rates vary if ambiguous sex estimate results are included or excluded. It could be argued that, when analysts judge a skeleton to be of “indeterminate” sex, it should be left out of calculations of sexing accuracy, since it is technically not an incorrect estimation. However, a counter‐argument can be made that if a trait, or suite of traits, produces a high quantity of “indeterminate sex” estimates, this may reveal important information about the degree of sexual dimorphism in a group, as well as the trait's usefulness; thus it should be reported. Moreover, different analysts may differ in how confidently they judge a skeleton to be sexable rather than indeterminate. In cases in which analysts report that a method for sex estimation achieves a very high accuracy, it is possible that they could simply be consigning any specimen about which there is any ambiguity to the “indeterminate” category and utilizing only the ones where male or female is given. In such a case, the accuracy may be artificially overstated; for someone trying to use the method to sex archaeological material, the key statistic is how many specimens are accurately sexed compared to ones which are either incorrectly sexed or left unsexed. In the interest of clarity, two rates will be reported here. One, termed “raw accuracy,” measures how many specimens were sexed accurately compared to all observations, including indeterminate and wrongly sexed ones; a second, termed “accuracy,” measures only how many were accurately sexed out of only those for which a sex estimation was achieved. This will be carried out for rates calculated for the pooled sexes, and the sexes individually. As there is an uneven number of males and females in the sample, weighted percentages will be used when presenting both overall (pooled male and female) accuracy rates.

## 4 RESULTS

### 4.1 Overall sex estimates

Table 4 outlines the number of females, probable females, unknown sex, probable males, and males identified by the macroscopic and aDNA estimates of sex. One individual could not be assigned a genetic sex because of poor aDNA preservation, and was excluded. In addition, three other individuals were excluded as they could not be macroscopically sexed because of a lack of traits.

**Table 4:** Number of females, probable females, indeterminate sex, probable males and males identified by macroscopic and aDNA estimates of sex

Method	Female	Probable female	Indeterminate	Probable male	Male	Unobservable
Macroscopic sex estimates	13	6	4	12	28	3
aDNA sex estimates	25	–	–	–	40	1

Accuracy rates were high for all three sets of traits used to estimate sex (Table 5). All of these rates are consistent with those in other tests of sex estimation methods on postmedieval and modern skeletal collections (see Table 1). For estimates derived from the combined skull and pelvis traits, accuracy was 97.7%, and raw accuracy was 95.6%. Only one individual was assessed as ambiguous (a young male). A McNemar test shows no systematic difference between the combined macroscopic estimate and the genetic estimate (*p* = .500, *n* = 47). Cohen's Kappa also shows an almost perfect level of agreement between the two methods (*k* = 0.903, *p* ≤ .001, *n* = 47).

**Table 5:** Accuracy (%) of sex estimates derived from os coxae traits, skull traits, and the combined os coxae and skull traits for the whole sample, females, and males

Skeletal area	Total correct	Total indeterminate	Total incorrect	Total	Raw % accuracy	% accuracy
Pooled sex						
Skull and os coxae	45	1	1	47	95.6	97.7
Os coxae	45	2	2	49	91.8	95.7
Skull	46	8	6	60	76.7	88.4
Females						
Skull and os coxae	15	1	1	17	88.2	93.7
Os coxae	16	1	2	19	89.0	84.2
Skull	15	2	5	22	68.2	75.0
Males						
Skull and os coxae	29	0	0	29	100	100
Os coxae	28	1	0	29	96.7	100
Skull	31	6	1	38	81.6	96.9

*Note*. Not all individuals had both the skull and os coxae so totals vary between elements.

The os coxae estimate had an accuracy rate of 95.7% and raw accuracy was still high at 91.8%, demonstrating low levels of ambiguous sex estimates from the os coxae. A McNemar test shows no systematic difference between the macroscopic os coxae result and the genetic results (*p =* .500, *n* = 49). Cohen's Kappa shows an almost perfect level of agreement between the two methods (*k* = 0.907, *p* ≤ .001, *n* = 49).

The accuracy of the skull was 88.4%. However, the raw accuracy of the skull was lower (76.7%) highlighting higher proportions of ambiguous estimates. This is a similar rate to that identified by Đuric et al. (2005) who also had many ambiguous sex skulls. A McNemar test shows no systematic difference between the macroscopic skull results and the genetic results (*p* = .219, *n* = 60). Cohen's Kappa shows that whereas the level of agreement between skull derived estimates and genetic estimates is lower than the combined and os coxae estimates, it is still substantial (*k* = 0.747, *p* ≤ .001, *n* = 60).

Table 5 presents the sex estimation results for the os coxae, skull, and combined skull and os coxae traits for males and females separately. In both cases the combined estimate performed best (males 100% and females 93.7%). The os coxae estimates (males 100% and females 84.2%) were more accurate than the cranial estimates (male 96.6% and females 75%). However, both the os coxae and cranial estimates were more accurate on males, especially the latter. It is worth pointing out here that for the one female incorrectly sexed as male using the os coxae, only a single Phenice trait was observable. This was significant because when at least two Phenice traits were observable, the accuracy in the identification of women was, like for men, 100%.

As the degree of sexual dimorphism changes with age, it was important to assess whether incorrectly assessed males and females were old or young. While the sample sizes were too small to test statistically, for women, no such effect was noted. One young female classified as male overall, whereas a young female, two middle aged females, and an old female classified as male by the skull. In males, while the os coxae was correct for all individuals, based on to the skull a young male was classified as female, and a further two young, two middle and one old male had a combination of male and female cranial traits.

### 4.2 Individual trait accuracy

Table 6 outlines the accuracy rates, McNemar and Cohen's Kappa results for each trait. Os coxae traits had higher accuracy and fewer ambiguous scores than cranial traits, with all but the preauricular sulcus scoring 80% or higher. For the Phenice traits, iliac shape, preauricular sulcus, arc compose, and subpubic angle accuracy rates accord well with published tests on known‐sex material (Đuric et al., 2005; Karsten, 2018; McFadden & Oxenham, 2015; Novak et al., 2012). In addition, Cohen's Kappa tests demonstrated that most os coxae traits had higher levels of agreement with the genetic sex estimates than cranial traits, with the Phenice traits, sciatic notch and subpubic angle having perfect or almost perfect agreement. The agreement between genetic estimates and the arc compose and the ilium shape was substantial, whereas the preauricular sulcus had moderate agreement. McNemar tests only identified a trend for a systematic difference between the genetic sex estimate for the arc compose.

**Table 6:** The total number of skull and os coxae traits observed, the numbers with correct, ambiguous and incorrect results and their accuracy rates

Trait	Total correct	Total?	Total incorrect	Total	Raw % accuracy	Total sexed	% accuracy	McNemar test	Cohen's kappa	*p*
Ventral arch	48	2	2	52	92.3	50	96.0	1.000	0.937	**<.001**
Ischiopubic ramus	43	5	0	48	89.6	43	100.0	1.000	1.000	**<.001**
Subpubic concavity	46	0	0	46	100.0	46	100.0	1.000	1.000	**<.001**
Sciatic notch	61	11	3	75	81.3	64	95.3	1.000	0.843	**<.001**
Arc compose	59	4	7	70	84.3	66	89.4	**0.063**	0.722	**<.001**
Preauricular sulcus	53	0	19	72	73.6	71	73.6	0.277	0.560	**<.001**
Subpubic angle	35	2	0	37	94.6	35	100.0	1.000	1.000	**<.001**
Ilium shape	49	3	10	62	79.0	59	83.1	0.687	0.671	**<.001**
Nuchal crest	34	11	9	54	62.9	43	79.1	0.180	0.471	**.001**
Mastoid process	66	28	10	104	63.5	76	86.8	1.000	0.741	**<.001**
Glabella	36	8	8	52	69.2	44	81.8	1.000	0.617	**<.001**
Supraoribital margin	55	19	20	94	57.9	75	72.4	0.581	0.377	**.013**
Frontal bossing	22	12	10	44	50.0	32	68.8	**0.007**	0.143	.299
Zygomatic arch extension	58	24	18	100	58.0	76	76.3	0.508	0.406	**.012**
Forehead inclination	30	5	9	44	68.2	39	76.9	0.508	0.494	**.002**
Mental eminence	40	14	1	55	72.7	41	97.6	1.000	0.951	**<.001**
Flaring at gonial angle	52	17	23	92	56.5	75	69.3	1.000	0.297	.060
Overall mandible shape	27	3	7	37	72.9	34	79.4	1.000	0.639	**<.001**

Key: Total? = total indeterminate. McNemar and Cohen's kappa tests were run without ambiguous scores. Significant or near significant values in bold.

Cranial trait accuracy rates are generally lower and more variable than the pelvic traits (cranial traits ranged from 68.8% to 97.6%). In addition, greater differences exist between accuracy and raw accuracy rates for cranial traits highlighting higher number of ambiguous estimates. McNemar tests showed that frontal bossing produced results systematically different to those obtained from genetics and Cohen's Kappa reveals that the only cranial trait with almost perfect agreement with genetic estimates was the mental eminence (see results in Table 6). The traits with substantial agreement were the mastoid processes, glabella, and overall mandible shape. The nuchal crest, zygomatic arch extension and frontal inclination showed moderate agreement, whereas gonial flaring and the supraorbital margin have only fair agreement. Frontal bossing performed badly with only slight agreement in estimates, unsurprising given the systematic differences in results to the aDNA tests.

Tables 7 and 8 present the accuracy for traits in males and female separately (see Tables 7 and 8) and the distribution of our scores according to male and female categories of the standards is presented in Table 9. The probability of someone from our sample scoring as female (and males by inference) for each score is presented in Table 9. For example, someone with a ventral arch score of 1 will have a probability of 1.0 for being female and therefore 0.0 for being male.

**Table 7:** Skull and os coxae traits observed, the totals with correct, ambiguous and incorrect results and their accuracy rates (%) for males

Trait	Score M	% score M	Score F	% Score F	Total sexed	Score?	% score?	Total	% raw accuracy
Ventral arch	27	93.1	2	6.5	29	2	6.5	31	87.1
Ischiopubic ramus	27	100.0	0	0.0	27	5	15.6	32	84.4
Subpubic concavity	27	100.0	0	0.0	27	0	0.0	27	100
Sciatic notch	36	94.7	2	4.6	38	6	13.6	44	81.2
Arc compose	41	100.0	0	0.0	41	0	0.0	41	100
Preauricular sulcus	29	69.2	13	30.8	42	0	0.0	42	69.0
Subpubic angle	17	100.0	0	0.0	17	2	10.5	19	89.5
Ilium shape	28	87.5	4	11.4	32	3	8.6	35	80
Nuchal crest	26	92.9	2	6.1	28	5	15.2	33	78.8
Mastoid process	40	90.9	4	6.6	44	17	27.9	61	65.6
Glabella	23	85.2	4	12.9	27	4	12.9	31	74.2
Supraoribital margin	34	64.2	19	29.2	53	12	18.5	65	52.3
Frontal bossing	11	57.9	8	29.6	19	8	29.6	27	40.7
Zygomatic arch extension	45	90.0	5	8.6	50	8	13.8	58	77.6
Forehead inclination	21	87.5	3	11.5	24	2	7.7	26	80.1
Mental eminence	22	95.7	1	3.0	23	10	30.3	33	66.7
Flaring at gonial angle	37	78.7	10	19.2	47	5	9.6	52	71.2
Overall mandible shape	14	82.4	3	15.8	17	2	10.5	19	73.7

Key: Score M = male, Score F = female, Score? = indeterminate. Accuracy is presented in % score M.

**Table 8:** Skull and os coxae traits observed, the totals with correct, ambiguous and incorrect results and their accuracy rates (%) for females

Trait	Score F	% Score F	Score M	% score male	Total sexed	Score?	% score?	Total	% raw accuracy
Ventral arch	21	100.0	0	0.0	21	0	0.0	21	100.0
Ischiopubic ramus	16	100.0	0	0.0	16	0	0.0	16	100.0
Subpubic concavity	19	100.0	0	0.0	19	0	0.0	19	100.0
Sciatic notch	25	96.2	1	3.9	26	5	19.2	31	80.6
Arc compose	18	72.0	7	28.0	25	4	16.0	29	62.1
Preauricular sulcus	24	84.2	6	15.8	30	0	0.0	30	80.0
Subpubic angle	18	100.0	0	0.0	18	0	0.0	18	100.0
Ilium shape	21	77.8	6	22.2	27	0	0.0	27	77.8
Nuchal crest	8	53.3	7	46.7	15	6	40.0	21	38.1
Mastoid process	26	81.3	6	18.8	32	11	34.4	43	60.5
Glabella	13	76.5	4	23.5	17	4	23.5	21	61.9
Supraoribital margin	21	75.0	7	25.0	28	10	35.7	38	55.3
Frontal bossing	11	84.6	2	15.4	13	4	30.8	17	64.7
Zygomatic arch extension	13	50.0	13	50.0	26	16	61.5	42	31.0
Forehead inclination	9	60.0	6	40.0	15	3	20.0	18	50.0
Mental eminence	18	100.0	0	0.0	18	4	22.2	22	81.8
Flaring at gonial angle	15	53.6	13	46.4	28	12	42.9	40	37.5
Overall mandible shape	13	76.5	4	23.5	17	1	5.9	18	72.2

Key: Score M = male, Score F = female, Score? = indeterminate. Accuracy is presented in the % Score F column.

**Table 9:** Trait by trait distribution by sex, and percentage probability of someone with that score being female

Trait	Score	1	2	3	4	5
Ventral arch	Female %	100.0	–	0.0	–	0.0
	Male %	5.0	–	10.0	–	85.0
	%female prob	0.95	–	0.00	–	0.00
Ischiopubic ramus	Female %	100.0	–	0.0	–	0.0
	Male %	0.0	–	19.0	–	81.0
	%female prob	1.00	–	0.00	–	0.00
Subpubic concavity	Female %	100.0	–	0.0	–	0.0
	Male %	0.0	–	0.0	–	100.0
	%female prob	1.00	–	0.00	–	0.00
Sciatic notch	Female %	26.3	52.6	15.8	0.0	5.3
	Male %	0.0	7.4	14.8	48.1	29.6
	%female prob	1.00	0.88	0.52	0.00	0.15
Arc compose	Female %	38.9	22.2	11.1	11.1	16.7
	Male %	0.0	0.0	0.0	4.3	95.7
	%female prob	1.00	1.00	1.00	0.72	0.15
Preauricular sulcus	Female %	15.8	21.1	5.3	42.1	15.8
	Male %	0.0	3.8	0.0	26.9	69.2
	%female prob	1.00	0.85	1.00	0.61	0.19
Subpubic angle	Female %	80.0	20.0	0.0	0.0	0.0
	Male %	0.0	0.0	8.3	8.3	83.3
	%female prob	1.00	1.00	0.00	0.00	0.00
Ilium shape	Female %	61.1	11.1	5.6	16.7	5.6
	Male %	0.0	8.7	13.0	17.4	60.9
	%female prob	1.00	0.56	0.30	0.49	0.08
Nuchal crest	Female %	19.0	14.3	33.3	33.3	0.0
	Male %	0.0	6.1	15.2	57.6	21.2
	%female prob	1.00	0.70	0.69	0.37	0.00
Mastoid process	Female %	13.6	45.5	27.3	13.6	0.0
	Male %	0.0	6.0	24.3	39.3	30.4
	%female prob	1.00	0.88	0.53	0.26	0.00
Glabella	Female %	19.0	42.9	19.0	19.0	0.0
	Male %	0.0	12.9	12.9	48.4	25.8
	%female prob	1.00	0.77	0.60	0.28	0.00
Supraoribital margin	Female %	9.5	42.9	23.8	23.8	0.0
	Male %	4.3	30.4	26.1	69.6	13.0
	%female prob	0.69	0.58	0.48	0.25	0.00
Frontal bossing	Female %	64.7	–	23.5	–	11.8
	Male %	29.6	–	29.4	–	40.7
	%female prob	0.69	–	0.44	–	0.22
Zygomatic arch extension	Female %	4.8	23.8	42.9	19.0	9.5
	Male %	6.7	3.3	16.7	40.0	33.3
	%female prob	0.42	0.88	0.72	0.32	0.22
Forehead inclination	Female %	50.0	–	16.7	–	33.3
	Male %	11.5	–	7.7	–	80.8
	%female prob	0.81	–	0.68	–	0.29
Mental eminence	Female %	18.2	63.6	18.2	0.0	0.0
	Male %	0.0	3.4	34.5	41.4	34.5
	%female prob	1.00	0.95	0.35	0.00	0.00
Flaring at gonial angle	Female %	9.5	28.6	28.6	33.3	0.0
	Male %	0.0	21.4	10.7	42.9	25.0
	%female prob	1.00	0.57	0.73	0.44	0.00
Overall mandible shape	Female %	72.2	–	5.6	–	22.2
	Male %	12.0	–	8.0	–	80.0
	%female prob	0.86	–	0.41	–	0.22

High accuracy rates for males and females were identified for the pubic traits, especially Phenice traits which are scored on a three‐point scale (see Tables 7 and 8). For these traits, the score distributions demonstrate that the probability that someone with a female score being female, and vice versa for males, is almost certain. A similar situation was observed for the sciatic notch, which also had high accuracy rates for men and women (94.5% males and 96.2% females), showing that it is a reliable indicator even though 14.8% of male and 15.8% of females scored ambiguous. However, the arc compose, preauricular sulcus, and ilium shape have sex differences in accuracy rates. The preauricular sulcus had a higher accuracy rate for females, with nearly all females scoring in the female categories (84.2%), whereas 30.8% of males scored in female categories resulting in a lower accuracy (69.2%). For the arc compose all males scored 4 or 5, giving a 100% accuracy, whereas one female scored ambiguous and 27.8% were in the male categories. A similar trend is observed for the ilium shape, with 20% of women being misclassified as male, as opposed to only 8.7% of males being classified as female. Ambiguous scores here were also more likely to be male.

For the nuchal crest, mastoids, forehead inclination, glabella, zygomatic arch extension, gonial flaring, and overall mandible shape we see similar trends whereby female accuracy is lower than male (see Tables 7 and 8). For all traits multiple females score in category 4 (Table 9). In addition, for the nuchal crest, glabella, zygomatic arch extension, and forehead inclination scores 3 were also more likely to be female (see Table 9). This suggests that for these traits St John's females are more robust than the population from which the method scores were derived. The opposite can be said for supraorbital margins and frontal bossing where male accuracy rates were lower than female accuracy rates (supraorbital margin 64.2% male, 75% female, frontal bossing 57.9% male, and 84.6% female). Only for the mental eminence did we see that an ambiguous score more frequently belonged to a male. For the former two traits we see few individuals scoring in the definitive categories “1” and “5,” but a tendency for scores in the middle three categories (2–4 for supraorbital margins and 3 for frontal bossing). This infers a lower level of sexual dimorphism in these traits generally as is highlighted by the differences in raw accuracy and accuracy rates.

## 5 DISCUSSION

Comparison of macroscopic and aDNA sex estimates demonstrate that methods derived from postmedieval and modern collections are similarly accurate when applied to the medieval skeletal collection from St John's Hospital, Cambridge. When considering the accuracy of the different macroscopic estimates, as with results obtained from tests on known‐sex material, those made from a combination of skull and os coxae traits slightly outperform the os coxae estimate alone, even though both had the same level of agreement with genetic estimates. Both estimates resulted in a low level of ambiguous cases, largely because of the excellent performance of the pubis traits. Although skull estimates do not have such a high level of accuracy and agreement with the genetic estimates, there was still a substantial level of agreement and 88.4% accuracy when ambiguous estimates were excluded. However, their use without the os coxae results in 13% of individuals remaining unsexed. This is a trend also observed by Đuric et al. (2005) where a similar number of crania (15%) could not be given an estimate. This demonstrates that cranial based estimates are valuable when the os coxae is not available, although their application alone will result in more individuals remaining unsexed. Overall, this research shows that these methods have been, and are appropriate for sex estimation on earlier material despite differences in lifestyle and nutrition.

While few sex estimates were incorrectly made, inhibiting a statistical analysis, completeness can be highlighted as an issue. As the pubis traits are most accurate, when they are observable sex can be accurately estimated, even if no other traits are observable. However, as the most accurate traits this means that when unobservable, more misclassifications are likely; os coxae accuracy rates for women appeared lower than for men as more pelves lacked some/all Phenice traits, and there were more misclassifications when only the skull was observable. As such, at least for this population, great care should be taken when pubic traits are unobservable, and to estimate sex it is necessary that other traits with high accuracies and agreement, for example, the sciatic notch, mastoids or the mental eminence, must be observable. When all these skeletal areas are all absent, one has to evaluate necessity of a sex estimate versus the error that it may introduce.

As it is not always possible to undertake aDNA analysis on all skeletal remains excavated, and the nature of the material means it is usually fragmented or incomplete, it is still desirable to improve the accuracy of macroscopic based methods. Two clear features emerged from our data. First, some traits had a sex bias in accuracy where either males or females were more accurately identified and second, some traits had a high quantity of ambiguous scores, as highlighted by differences in accuracy and raw accuracy rates, or had significant score overlap. Both issues result from differences in sexual dimorphism between our sample and that used to create the sex estimation methods. By presenting the distribution of scores for individual traits it was possible to see the degree of trait sex dimorphism in our sample and how it relates to the scores and cut off points for the methods.

Os coxae traits were in general much more accurate than those of the cranium. Considering the individual os coxae traits first, generally there were low levels of ambiguous scores. Pubic traits and the sciatic notch performed very well on our sample, and score distributions matched well with the standards. However, as can be inferred from lower McNemar test scores, the arc compose, Ilium shape, and preauricular sulcus score distribution differed to that of the standards. For the arc compose and iliac shape, many females scored in male categories ultimately resulting in a lower accuracy for women. For the preauricular sulcus the opposite trend is observed, where nearly all women were correctly identified in scores 1–4, but 31% of males were misclassified. For the arc compose and the preauricular sulcus it is possible to reconsider Score 4 as ambiguous with Score 3 being reclassified as female. As the distribution of iliac shape scores was not as skewed, it would be beneficial to look at a larger sample size before changing cut‐off values.

The lower accuracies of the arc compose and preauricular sulcus is unsurprising as both have been shown accurate (Arsuaga, Lorenzo, & Carretero, 1995; Đuric et al., 2005) and inaccurate in tests on known‐sex groups (Arsuaga et al., 1995; Karsten, 2018; Novak et al., 2012). Đuric et al. (2005) found over 90% accuracy for the arc compose, however they only assessed males. Our study supports their findings, but we show it is unsuitable here for St John's females, and therefore problematic for sex estimation. Our men had a higher percentage of sulci than reported by Đuric et al. (2005) and Novak et al. (2012), which resulted in lower accuracy for St John's men. This differs to Novak et al. (2012) who had better accuracy in women. While this issue may stem from interobserver error, there is some debate surrounding the relationship between the preauricular sulcus and sex. Radiographic studies have shown that it is usually present in fewer than 25% of women (Dee, 1981; Gülekon & Turgut, 2001; Spring, Lovejoy, Bender, & Duerr, 1989) with MacLaughlin and Cox (1989) suggesting preauricular sulcus presence is related to pelvic size as well as morphology and childbirth. Without knowing the size of the men and women in the published studies we are not able to study this further, but population size differences may be a factor. Thus at present its use as a sex indicator could be questioned.

Unlike the os coxae, where variation between male and female morphology relate to reproductive function and have been selected for (Schwartz, 2007), cranial dimorphism relates more strongly to differences in body size and musculature (Mays & Cox, 2000), which can be influenced by social patterns of physical activity, sexual selection, disease, or dietary habits. As such, levels of cranial sexual dimorphism have a greater potential to vary between groups. In cranial traits we also saw sex biases. For five traits (nuchal crest, glabella, zygomatic arch extensions, forehead inclination, and gonial flaring) Score 3 was more likely to be female than male. Furthermore, for all cranial traits except the mental eminence, a high proportion of females (between 13.6% and 33.3%) scored 4 or 5. This explained the lower cranial estimate accuracy in females. Unlike the os coxae traits where there were few ambiguous estimates, the cranial traits raw accuracy rates demonstrate clearly the number of ambiguous scores. There is also significant overlap in scores between males and females for mastoid processes, frontal bossing, and the supraorbital margins. Overall, this highlights the importance of assessing accuracy rates separately for males and females, and not relying solely on pooled sex accuracy rates, which can be deceptive.

Skewing in score distributions and/or high ambiguous rates reflect the possibility that for medieval English people, robusticity/development and biological sex relate differently than in modern reference populations; most notably as the females from St John score frequently in ambiguous or Score 4, they are more robust than the modern females used to create the standards. This could result from the fact that medieval women often performed hard physical work; this is particularly likely for poor women such as those likely to be buried in a charitable institution such as the Hospital (see Cessford, 2015). In addition, diet was probably coarser, although we would expect males to more consistently score higher too. Like the os coxae traits, accuracy can be improved for this population by reconsidering the boundaries for “male” and “female.” In traits where females were more robust than standards would imply (the nuchal crest, gonial flaring, and zygomatic arch extensions) we can acknowledge that Score 3 likely represents female and Score 4 could be either male or female. In a similar fashion the reverse can be done for the supraorbital margins, where males scored frequently as 3 and occasionally 2.

Many researchers highlight the importance of considering the age of an individual before giving an overall sex estimate (Mays & Cox, 2000). As the development of “masculine” features is dependent on an extended growth period and increased muscularity, males who die in young adulthood may still appear “feminine.” Conversely, as females age, their skeletal traits can become more robust especially of the skull (Buikstra & Ubelaker, 1994). In this research very few individuals were incorrectly sexed which prevented analysis of whether this was a factor in our misclassifications, but one young male was assessed as female from cranial traits, with a further two scored as ambiguous. Again, this reinforces the need to be careful when assessing young adults. The females that were misclassified by either combined or individual elements, were of different ages. However, it would be useful to examine more female individuals as the sample size for some age groups was not sufficiently large to be certain of the result.

Going forward it would be valuable to know which combination of traits produced the highest accuracy rates. Unfortunately, for some traits our sample size was limited preventing detailed statistical analysis (e.g., discriminant functions). It would also be valuable to test whether other medieval populations, or those that differ significantly in lifestyle to those used in the creation of sex estimation methods, have similar trait score distributions and sexual dimorphism to that identified in the St John's individuals, or if it is idiosyncratic to the group. We have to bear in mind that the sample used here represents individuals from a hospital cemetery, which may incur some biases. Given that sexual dimorphism is significantly affected by various social and environmental factors (Ubelaker & DeGaglia, 2017), comparison with contemporaneous collections of different social backgrounds, geographical locations, and time periods could be highly enlightening in terms of both understanding sexual dimorphism in commonly assessed traits, and their appropriateness and usefulness in assessing sex in archaeological skeletal collections.

## 6 CONCLUSIONS

This research examined whether commonly used macroscopic traits for sex estimation produced accuracy rates similar to those achieved on known‐sex postmedieval and modern material when assessed in a sample of medieval skeletons from the Hospital of St John the Evangelist, Cambridge. Through a comparison of macroscopic to genetic data, this research shows that estimates derived from a combination of skull and pelvic traits, and the pelvis and the skull individually, were similarly accurate to tests on known‐sex postmedieval and modern remains, demonstrating their reliability for our sample. Furthermore, like other studies, the pelvic traits outperformed those of the skull. Individually, traits showed differences in the degree and scaling of sexual dimorphism between the medieval sample and that of the original collections used to create the standards, especially for skull traits. Here, greater robusticity in females and a lower degree of dimorphism resulted in low accuracy rates. This knowledge can now be used to improve the accuracy of sex estimates for the St John's material through reanalysis of the scores ascribed to “male” and “female,” as well as the removal of the preauricular sulcus and arc compose from analysis. Following this, and the varying results published in literature, the results indicate that researchers using macroscopic assessment alone for sex estimation should reconsider the use of the arc compose and preauricular sulcus in their analysis. In addition, it is wise not to base sex estimates on a few cranial traits, especially if the degree of sexual dimorphism for a population is not known. Future research should test whether these new standards are more accurate on other material from medieval Cambridge, and explore other populations with differing social and environmental backgrounds.

## Supporting information


**Supplementary graph 1** Value of ratio of reads mapping to Y vs. X + Y (Ry) for each individual. Standard error bars in red.

**Supplementary graph 2** Ranges of Ry for assignments XX (female), XY (male), and XX** (consistent with XY but not XX). Whisker range is interquartile ranges

**Supplementary table 1** Contamination data, genetic sex and biological sex estimates for the St John's hospital sample. Results are either returned as XX (female) or XY (male), (“XX” when Ry + CI [=1.96*SE] < 0.016 and “XY” when Ry‐ CI >0.075), “consistent with XX but not XY” (RY‐ CI < 0.016 and Ry + CI < 0.075), “consistent with XY but not XX” (RY‐ CI > 0.016 and Ry + CI > 0.075), else “Not Assigned. No = Skeleton number. M = Male,?M = probable male,? = undetermined,?F = probable female, F = female. U = unobservable. ND = not determinable

## Footnotes

1 We are aware that diagnosis of sex from aDNA on the basis of Y chromosome detection may not necessarily reflect biological (anatomical, hormonal) or social “sex,” and that the latter may not necessarily always take discreet forms of “male” or “female (Geller, 2009). Such discrepancies, however, are statistically uncommon, and in this article we use genetic sex based upon aDNA analysis as a proxy for a bottom‐line correct determination of biological sex. Any analysis of the matter potentially incurs such issues and requires a similar assumption; for instance, analyses of historically “known‐sex” samples routinely assume that hospital or autopsy records accurately reflect the biological sex of each individual.
