# Supplementary figures and images for: Evaluating macroscopic sex estimation methods using genetically sexed archaeological material: The medieval skeletal collection from St John's Divinity School, Cambridge
Source: Am J Phys Anthropol. 2018 Dec 21;168(2):340–51. doi: 10.1002/ajpa.23753 (PMC6492084; doi:10.1002/ajpa.23753)

Ratio of reads mapping to Y vs. X + Y chromosomes ( $R_y$ )

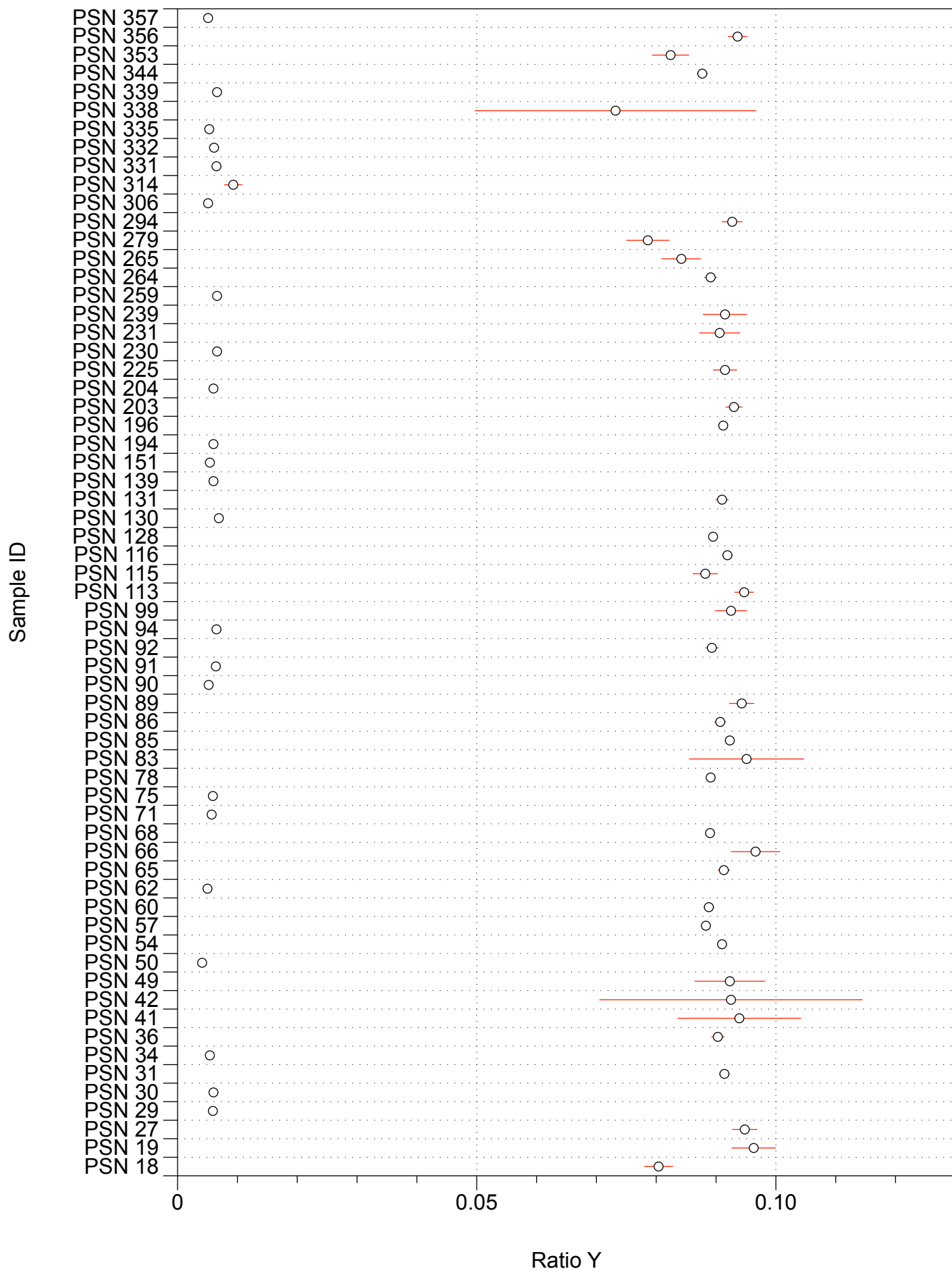

Supplement: Supplementary file 1 — Supplementary graph 1 Value of ratio of reads mapping to Y vs. X + Y (Ry) for each individual. Standard error bars in red. [file AJPA-168-340-s001.pdf]

Ranges of  $R_y$  values for assignments

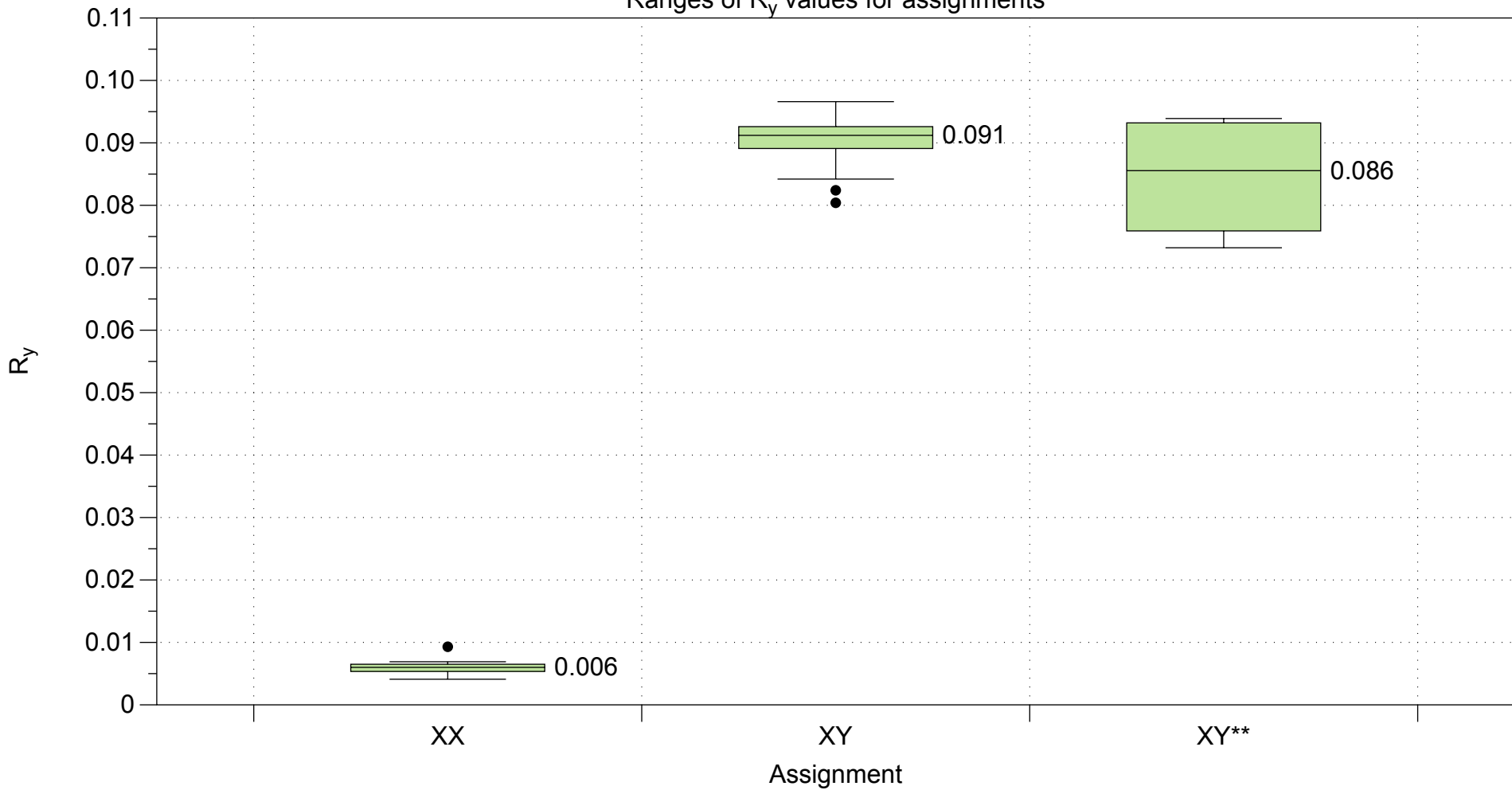

Supplement: Supplementary file 2 — Supplementary graph 2 Ranges of Ry for assignments XX (female), XY (male), and XX** (consistent with XY but not XX). Whisker range is interquartile ranges [file AJPA-168-340-s002.pdf]
