# Supplementary material for: Evaluating macroscopic sex estimation methods using genetically sexed archaeological material: The medieval skeletal collection from St John's Divinity School, Cambridge
Source: Am J Phys Anthropol. 2018 Dec 21;168(2):340–51. doi: 10.1002/ajpa.23753 (PMC6492084; doi:10.1002/ajpa.23753)
Supplement: Supplementary file 3 — Supplementary table 1 Contamination data, genetic sex and biological sex estimates for the St John's hospital sample. Results are either returned as XX (female) or XY (male), (“XX” when Ry + CI [=1.96*SE] < 0.016 and “XY” when Ry‐ CI >0.075), “consistent with XX but not XY” (RY‐ CI < 0.016 and Ry + CI < 0.075), “consistent with XY but not XX” (RY‐ CI > 0.016 and Ry + CI > 0.075), else “Not Assigned. No = Skeleton number. M = Male,?M = probable male,? = undetermined,?F = probable female, F = female. U = unobservable. ND = not determinable [file AJPA-168-340-s003.docx]

| **PSN** | **No** | **Raw Reads** | **Aligned to hg19** | **Duplicates** | **% Duplicate** | **% Unique Human** | **Cov (x)** | **mtDNA Contamination Estimate** | **Genetic Sex** | **R_y_** | **Macroscopic Sex** |
| --- | --- | --- | --- | --- | --- | --- | --- | --- | --- | --- | --- |
| 29 | 1264 | 36,114,839 | 1,305,623 | 14,601 | 1.12% | 3.57% | 0.035 | 0.17% | F | 0.0059 | F |
| 30 | 1231 | 34,097,666 | 214,327 | 2,080 | 0.97% | 0.62% | 0.006 | 0.00% | F | 0.0060 | F |
| 34 | 2145 | 46,666,029 | 10,836,230 | 164,530 | 1.52% | 22.87% | 0.286 | 0.61% | F | 0.0054 | ? |
| 50 | 2194 | 55,611,453 | 35,607,452 | 736,439 | 2.07% | 62.70% | 0.934 | 0.83% | F | 0.0041 | F |
| 62 | 2220 | 49,607,914 | 6,396,880 | 90,590 | 1.42% | 12.71% | 0.169 | 0.83% | F | 0.0050 | F |
| 71 | 2248 | 32,418,717 | 2,808,215 | 35,813 | 1.28% | 8.55% | 0.074 | 0.45% | F | 0.0057 | F |
| 75 | 2255 | 26,768,775 | 5,060,091 | 144,789 | 2.86% | 18.36% | 0.132 | 0.22% | F | 0.0059 | ?F |
| 90 | 3183 | 38,629,951 | 192,404 | 1,876 | 0.98% | 0.49% | 0.005 | 0.00% | F | 0.0052 | F |
| 91 | 3186 | 42,987,172 | 8,166,149 | 138,458 | 1.70% | 18.67% | 0.215 | 0.51% | F | 0.0064 | ?F |
| 94 | 3207 | 27,230,106 | 1,776,000 | 19,769 | 1.11% | 6.45% | 0.047 | 0.28% | F | 0.0065 | F |
| 130 | 5059 | 47,569,229 | 424,471 | 6,467 | 1.52% | 0.88% | 0.011 | 0.00% | F | 0.0069 | ? |
| 139 | 5093 | 39,309,953 | 282,847 | 4,298 | 1.52% | 0.71% | 0.007 | ND | F | 0.0060 | M |
| 151 | 5110 | 41,911,316 | 17,369,335 | 294,833 | 1.70% | 40.74% | 0.457 | 0.50% | F | 0.0054 | M |
| 194 | 1241 | 24,060,425 | 230,314 | 3,170 | 1.38% | 0.94% | 0.006 | 0.31% | F | 0.0060 | F |
| 204 | 1279 | 39,223,415 | 1,851,774 | 30,028 | 1.62% | 4.64% | 0.049 | 0.36% | F | 0.0060 | F |
| 230 | 1341 | 32,743,414 | 1,578,058 | 21,928 | 1.39% | 4.75% | 0.042 | 2.11% | F | 0.0066 | F |
| 259 | 1414 | 34,094,842 | 2,709,597 | 33,628 | 1.24% | 7.85% | 0.072 | ND | F | 0.0066 | F |
| 306 | 1508 | 45,091,878 | 10,478,239 | 206,953 | 1.98% | 22.78% | 0.275 | 0.35% | F | 0.0051 | F |
| 314 | 1525 | 3,942,958 | 94,909 | 1,257 | 1.32% | 2.38% | 0.003 | 1.72% | F | 0.0093 | F |
| 331 | 3297 | 28,458,780 | 326,261 | 2,950 | 0.90% | 1.14% | 0.009 | 0.65% | F | 0.0065 | F |
| 332 | 3299 | 29,852,474 | 5,786,137 | 188,396 | 3.26% | 18.75% | 0.15 | 0.16% | F | 0.0061 | M |
| 335 | 3310 | 26,542,191 | 13,334,397 | 204,630 | 1.53% | 49.47% | 0.352 | 0.45% | F | 0.0053 | F |
| 339 | 3347 | 27,823,306 | 440,634 | 4,281 | 0.97% | 1.57% | 0.012 | 2.35% | F | 0.0066 | F |
| 357 | 3531 | 32,398,011 | 11,583,284 | 199,199 | 1.72% | 35.14% | 0.305 | 1.79% | F | 0.0051 | F |
| 404 | 4097 | 36,931,952 | 12,177,935 | 184,840 | 1.52% | 32.47% | 0.321 | 1.67% | F | 0.0047 | F |
| 18 | 1210 | 50,845,300 | 501,170 | 4,948 | 0.99% | 0.98% | 0.013 | 0.56% | M | 0.0804 | M |
| 19 | 1211 | 38,021,584 | 240,079 | 3,394 | 1.41% | 0.62% | 0.006 | 0.20% | M | 0.0963 | M |
| 27 | 1220 | 36,887,918 | 721,244 | 7,918 | 1.10% | 1.93% | 0.019 | 0.00% | M | 0.0948 | ?M |
| 31 | 1232 | 22,538,026 | 3,967,506 | 63,685 | 1.61% | 17.32% | 0.105 | 1.30% | M | 0.0914 | M |
| 36 | 2150 | 19,544,649 | 2,564,954 | 45,056 | 1.76% | 12.89% | 0.067 | 0.76% | M | 0.0903 | M |
| 41 | 2176 | 33,046,813 | 29,178 | 244 | 0.84% | 0.09% | 0.001 | 0.00% | M | 0.0939 | M |
| 49 | 2192 | 34,066,868 | 99,712 | 1,967 | 1.97% | 0.29% | 0.003 | 0.83% | M | 0.0923 | M |
| 54 | 2203 | 35,042,981 | 4,885,284 | 82,007 | 1.68% | 13.71% | 0.129 | 0.30% | M | 0.0910 | M |
| 57 | 2209 | 36,906,931 | 24,489,602 | 435,964 | 1.78% | 65.17% | 0.644 | 0.92% | M | 0.0883 | M |
| 60 | 2215 | 36,830,838 | 4,203,835 | 71,780 | 1.71% | 11.22% | 0.111 | 0.36% | M | 0.0888 | ?M |
| 65 | 2228 | 32,504,979 | 2,797,165 | 36,824 | 1.32% | 8.49% | 0.074 | 0.63% | M | 0.0913 | M |
| 66 | 2230 | 34,466,383 | 194,269 | 3,142 | 1.62% | 0.55% | 0.005 | 0.00% | M | 0.0966 | M |
| 68 | 2239 | 32,210,759 | 6,877,520 | 104,311 | 1.52% | 21.03% | 0.181 | 0.35% | M | 0.0890 | M |
| 78 | 2260 | 31,387,977 | 5,072,853 | 80,319 | 1.58% | 15.91% | 0.134 | 0.44% | M | 0.0891 | M |
| 83 | 2273 | 30,815,989 | 38,766 | 2,776 | 7.16% | 0.12% | 0.001 | ND | M | 0.0951 | U |
| 85 | 2280 | 32,508,872 | 7,221,781 | 126,207 | 1.75% | 21.83% | 0.19 | 1.21% | M | 0.0923 | M |
| 86 | 3167 | 35,603,545 | 3,294,398 | 43,098 | 1.31% | 9.13% | 0.087 | 1.55% | M | 0.0907 | ?M |
| 89 | 3199 | 31,668,354 | 727,073 | 8,782 | 1.21% | 2.27% | 0.019 | 0.21% | M | 0.0943 | M |
| 92 | 3192 | 37,803,828 | 2,709,597 | 33,628 | 1.24% | 7.08% | 0.072 | 0.47% | M | 0.0893 | ?M |
| 99 | 3220 | 32,460,912 | 431,135 | 5,087 | 1.18% | 1.31% | 0.011 | 1.61% | M | 0.0925 | M |
| 113 | 3275 | 41,258,662 | 1,263,029 | 23,609 | 1.87% | 3.00% | 0.033 | 0.00% | M | 0.0947 | M |
| 115 | 3285 | 34,383,321 | 668,042 | 6,661 | 1.00% | 1.92% | 0.018 | 0.62% | M | 0.0882 | M |
| 116 | 3288 | 22,104,173 | 4,090,278 | 143,254 | 3.50% | 17.86% | 0.106 | 0.52% | M | 0.0919 | M |
| 128 | 5041 | 23,592,460 | 8,723,089 | 392,876 | 4.50% | 35.31% | 0.223 | 0.00% | M | 0.0895 | Und |
| 131 | 5064 | 32,834,626 | 2,607,507 | 39,563 | 1.52% | 7.82% | 0.069 | 1.54% | M | 0.0910 | ? |
| 196 | 1243 | 73,222,554 | 23,179,080 | 507,934 | 2.11% | 32.32% | 0.634 | 0.64% | M | 0.0912 | F |
| 203 | 1277 | 49,082,182 | 1,610,138 | 19,524 | 1.21% | 3.24% | 0.043 | 1.55% | M | 0.0930 | M |
| 225 | 1322 | 38,939,976 | 789,813 | 8,730 | 1.11% | 2.01% | 0.021 | 0.41% | M | 0.0915 | M |
| 231 | 1343 | 37,539,303 | 274,539 | 3,915 | 1.43% | 0.72% | 0.007 | ND | M | 0.0906 | ? |
| 239 | 1361 | 48,211,628 | 228,290 | 2,382 | 1.04% | 0.47% | 0.006 | 0.00% | M | 0.0915 | M |
| 264 | 1433 | 7,727,115 | 2,550,954 | 38,122 | 1.49% | 32.52% | 0.067 | 1.89% | M | 0.0891 | M |
| 265 | 1443 | 48,689,291 | 272,531 | 4,090 | 1.50% | 0.55% | 0.007 | ND | M | 0.0842 | M |
| 294 | 1485 | 43,235,367 | 1,233,241 | 20,569 | 1.67% | 2.80% | 0.032 | ND | M | 0.0927 | ?F |
| 344 | 3369 | 34,789,140 | 3,442,338 | 46,549 | 1.35% | 9.76% | 0.091 | 0.29% | M | 0.0877 | M |
| 356 | 3475 | 23,484,281 | 1,137,032 | 15,675 | 1.38% | 4.77% | 0.03 | 0.00% | M | 0.0936 | M |
| 438 | 2094.1 | 33,574,161 | 1,588,268 | 23,674 | 1.49% | 4.66% | 0.042 | 0.27% | M | 0.0900 | ? |
| 51 | 2196 | 22,463,212 | 78,887 | 3,912 | 4.96% | 0.33% | 0.002 | 0.54% | U | 0.0644 | F |
| 279 | 2317 | 43,756,761 | 211,565 | 2,360 | 1.12% | 0.48% | 0.006 | ND | ?M | 0.0786 | M |
| 338 | 3318 | 12,140,444 | 5,150 | 32 | 0.62% | 0.04% | 0 | 4.43% | ?M | 0.0732 | F |
| 353 | 3398 | 33,492,923 | 288,094 | 3,424 | 1.19% | 0.85% | 0.008 | 0.30% | ?M | 0.0824 | M |
| 42 | 2178 | 30,987,670 | 6,837 | 96 | 1.40% | 0.02% | 0 | ND | ?M | 0.0925 | ? |

Supplementary table 1. Contamination data, genetic sex and biological sex estimates for the St John’s hospital sample. Results are either returned as XX (female) or XY (male), ("XX" when Ry+ CI (=1.96*SE) < 0.016 and "XY" when Ry- CI >0.075), "consistent with XX but not XY" (RY- CI < 0.016 and Ry+ CI < 0.075), "consistent with XY but not XX" (RY- CI > 0.016 and Ry+ CI > 0.075), else "Not Assigned. No= Skeleton number. M= Male, ?M= probable male, ?=undetermined, ?F=probable female, F=female. U=unobservable. ND= not determinable.
